# Relational security: conceptualization and operationalization in small-scale, strengths-based, community-embedded youth justice facilities

**DOI:** 10.1186/s13034-023-00638-3

**Published:** 2023-08-17

**Authors:** Fleur Souverein, Eva Mulder, Lieke van Domburgh, Arne Popma

**Affiliations:** 1 Child and Adolescent Psychiatry & Psychosocial Care department Amsterdam UMC, Meibergdreef 5, Amsterdam, 1105 AZ The Netherlands; 2 Amsterdam Public Health, Mental Health, Van der Boechorststraat 7, Amsterdam, 1081 BT The Netherlands; 3 Academische Werkplaats Risicojeugd, Postbus 53, Nijmegen, 6500 AB The Netherlands; 4 Child development and education, University of Amsterdam, Amsterdam, The Netherlands; 5 LUMC Curium – Child and Adolescent Psychiatry, Leiden University Medical Center, Leiden, The Netherlands

**Keywords:** Prison, Dynamic security, Institutional violence, Youth development, Youth justice, Forensic youth care, Justice-involved young people, Relational security

## Abstract

**Background:**

Given the developmental vulnerability of justice-involved youth, providing a safe environment in secure facilities is a paramount, yet challenging task. Within this complexity, a sound security framework is key. The security framework exists on three dimensions: physical, procedural and relational security. Existing knowledge points at the importance of a shift in focus on physical and procedural security towards relational security as the core of the security framework. At the same time there is a dearth of knowledge on relational security, particularly in the context of youth justice. This paper explores relational security and its working mechanisms in practice.

**Methods:**

This paper draws on findings of a comprehensive three-year evaluation of three small-scale, community-embedded facilities that are grounded in relational security. The approach of the evaluation was derived from action research, involving a cyclic process alternating between action, research and critical reflection, while engaging all stakeholders in the research process. The action research cycle involved qualitative research (a total of 63 semi-structured interviews) incorporating the perspective of staff, youth and parents.

**Results:**

Relational security is grounded in three distinct, but interrelated, elements – staff’s basic attitude, a constructive alliance between staff and youth, staff presence – and promotes a safe and therapeutic environment through several mechanisms.

**Conclusions:**

Relational security can be defined in a practical conceptualization; outlining a way of working that guides staff in how to establish a safe *and* therapeutic environment in secure facilities. This conceptualization finds support in the well-established literature covering the therapeutic alliance and can be substantiated by two aligning theories concerning youth justice strategies: social-ecological theory and self-determination theory. Relational security is not only *a way of working*, but also *a way of being.* It encompasses a vision about security and mentality towards justice-involved youth that sees them not merely as ‘risks to be managed’, but primarly as ‘resources to be developed’.

## Background

Justice-involved youth can be remanded or sentenced to a secure facility as a last resort [1]. Both from a child rights as well as scientific perspective these residential settings should be safe places where youth’s developmental needs are met, strengths and protective factors are built upon, and positive community connections are forged. Given the developmental vulnerability of justice-involved youth, providing a safe therapeutic environment is a paramount, yet challenging task. Youth in these facilities display a large variety of neurobiological, psychological and social problems [2]. They often have a history of abuse and other traumatic experiences triggering complex emotional needs [3]. Staff needs to handle externalizing behavior, like non-compliance and aggression, on one hand and internalizing behavior, like self-harm and suicide, on the other [4]. Within this complexity, a sound security framework is key.

The security framework is a systemic model, which can be defined by three distinct, but inter-related, security dimensions [5]: physical security, procedural security and relational security (also referred to as dynamic security). Physical security can be separated into measures within the physical design and construction of the institution (e.g. fences, locks) and the equipment that is available to staff to control the environment (e.g. alarms, camera’s). Procedural security includes the institutions policies and procedures that are in order to restrict and control communication, possessions, visits and movement; the protocols and instruments for risk and crisis management; and covers policies and procedures concerning service quality and governance. Definitions of relational security refer to the ability of staff to establish safety through their understanding of the context and each incarcerated individual, the ability to translate this information into appropriate actions and the quality of the relationship between staff and people incarcerated [e.g. 5, 6, 7]. Relational security has been labeled as ‘the best security element in any custodial setting’ [8: page. 233] or ‘the most valuable and unobtrusive form of control’ [9: page. 234]. There is, however, a dearth of research on the conceptualization of relational security, particularly in youth justice settings. This paper therefore explores the concept and its working mechanisms in practice. We do so within the context of small-scale, community-embedded youth justice facilities that are grounded in relational security.

Relational security finds strong support in the well-established literature covering the therapeutic alliance: across youth justice settings a constructive and collaborative therapeutic alliance is crucial for promoting positive youth development and preventing recidivism [e.g. 10, 11]. Relational security concerns actively utilizing this alliance to promote safety at individual and institutional level [12]. For example, the mere presence of staff and their sensitivity to things going on at the unit is an important factor related to safety [13]. Also, when staff serve as positive role models with the capacity to diplomatically and respectfully resolve conflicts, this discourages youth from engaging in aggressive behavior [14]. Support is further found in methods, like Non-Violent Resistance [NVR; 15], that aim promoting safety by actively (re)building constructive relationships between staff and youth and by diverting from repressive and restrictive approaches. Training staff in NVR leads to a decrease in violence and promotes a therapeutic institutional climate in secure youth facilities, irrespective of youth’s gender, age and intellectual abilities [15, 16]. Methods like NVR may provide a clear set of working processes to consistently provide a relational security approach in youth justice facilities.

Conventional prison-like youth justice settings, however, generally exert a high level of control through physical and procedural security. The downside of this security approach is that it leads to isolation from society, limited educational and job opportunities, limited contact with family and peers, limited physical movement, and limited autonomy. While all of these aspects are crucial for adolescent development and prevention of recidivism [17]. Also, in these settings youth may be subjected to coercive and aggressive measures, like seclusion or physical restraint. These measures are potentially traumatizing for youth, diminish youth’s treatment motivation, and disrupt constructive relationships between youth and staff; thereby interfering with the therapeutic process [18, 19]. Hence the nature of those secure settings, especially when combined with other adverse childhood experiences, can impair youth’s physical and mental health and their neurological, cognitive and social development [20]. This security approach may also have adverse effects on staff. Staff exerting restraint and seclusion report distressing emotions of uneasiness, fear, anxiety and guilt and can become mentally exhausted from the psychological strain [21]. Which in turn impacts their ability to appropriately attend to youth’s needs [22]. An overreliance on physical and procedural security may put the institution in a state of hypervigilance, as under greater restriction the risk of another incident increases [8]. This can be described as the ‘aggression-coercion cycle’: coercive security measures may protect staff and youth from any direct further damage, however, in the long run these measures are likely to exacerbate aggression in youth as well as in staff [23]. Thereby amplifying violence committed *by* and *against* youth. For all these reasons youth justice settings employing a predominantly repressive and risk-focused approach with a strong reliance on physical and procedural security foster, rather than curb, youth offending behavior [24].

While all three security dimensions need to be in place, existing knowledge does points at the importance of a shift from a prime focus on physical and procedural security towards relational security. At the same time some gaps in the literature appear. Several conceptualizations of relational security exist, all referencing similar phenomena, however, without going into depth and describing how these aspects interact and complement each other. The complexity of relational security in theory and practice is not well-defined. Publications on relational security further mostly emanate from conventional secure settings, which do not have relational security at the core of the security framework. There is a need to systematically examine how relational security operates in facilities that are foremost reliant on relational security. As a further matter existing literature almost exclusively emerged in adult forensic settings. It is important to make this contextual distinction as the underlying processes and manifestations of relational security may differ in a youth justice setting, given the unique developmental needs of youth. For example, as adolescence is characterized by a need for autonomy and independence from adult-influences, it makes youth particularly sensitive to authoritarian and controlling staff behavior [25]. This calls for research on relational security in youth justice settings.

This paper aims to contribute to filling this dearth of knowledge. We do so on the basis of a comprehensive three-year evaluation of three youth justice facilities that are grounded in relational security. Through action research the evaluation provided the unique opportunity to closely follow the development and implementation of these facilities; including a consensus building process and constant learning curve between practitioners and policy makers in developing this way of working. Within this context the current study explores two research questions incorporating the perspective of both unit staff and management as well as youth and their parents: How is relational security in a youth justice setting conceptualized?; and How does relational security contribute to safety in a youth justice setting?

## Method

### Setting

This study was conducted as part of a three-year evaluation project initiated by the Dutch Ministry of Justice to examine the feasibility and potential efficacy of an alternative custodial model: small-scale, community-embedded and grounded in relational security. In 2016, pilot sites were opened in three large cities in the Netherlands: Nijmegen (November 2016 – December 2017), Groningen (December 2016 – December 2017), and Amsterdam (September 2016 – December 2019). These small-scale facilities each accommodated eight youth, pre and post-trial, following the same vision and practical framework. Souverein and colleagues outline this framework and key operational elements [26, 27]. To describe the setting we outline the security framework of the facilities (as this is the main topic of this paper), which includes the procedures for screening and placement. We refer to aforementioned other publication by Souverein and colleagues for a detailed description of other operational elements. The facilities were classified as a high security setting in terms of relational security combined with low levels of physical and procedural security. The business case specified that relational security implies that staff relies on the relationships between staff and youth to ensure security [28]. A more concrete conceptualization of relational security was not defined on paper. Being pilot facilities, one of the aims was to develop a good understanding of the concept and practice of relational security, and how it ensures safety.

With regards to physical security, staff were trained to refrain from physical restraint and seclusion. Alarm pagers, high surrounding walls, and window fences also had no place in these facilities. The internal and external structure were designed to reflect a homely atmosphere. The few physical security measures in place were: 24 h camera supervision (the cameras were positioned as inconspicuously as possible); the main entrance of the facility was locked 24 h; youth were locked in their room during the night; and the windows in youth’s room were able to open only to a certain extent.

As part of procedural security, before placement there was a thorough screening and indication process; selecting youth who are appropriately matched to the level of security (e.g. youth who are not likely to abscond) and who can profit from the opportunities the community-embedded facility offers (i.e., continuation or initiation of protective factors in the community). Youth were only placed if professionals in the youth justice chain agreed the facility was an appropriate match to youth’s needs and youth expressed motivation to cooperate with staff and comply to the rules. This also meant youth could be transferred to facilities with higher levels of physical and procedural security as an ultimate consequence after rule-breaking. In addition a few other procedural measures could be applied: drugs testing and room searching, but only on the basis of a probable cause; use of mobile phones was prohibited inside the facility (when youth went on leave they were allowed to carry their mobile phone); and a hand held metal detector could be applied when youth came back from leave (but no invasive searches). Further, neither youth or staff wore an uniform; the facility was designed with ‘normal’ furniture instead of furniture specifically designed for a secure environment; and regular cutlery, instead of plastic cutlery, could be used by youth uncontrolled. Youth were able to move independently trough the facility during the day with free access to their own room and the communal areas. The facilities were located within a neighborhood, in close proximity to youth’s home environment; daytime activities, such as school or work, were organized outside the facility, to which youth were allowed to travel independently. The procedures for visitors were set up with few restrictions (i.e. no fixed visiting hours and no visitation). In any case the security measures in the facility allowed flexibility to be tailored to each individual youth’s needs at any given time. For each youth an individual case plan was drawn up to specify their security and care arrangements.

### Action research

The approach of the evaluation was derived from action research, involving a cyclic process alternating between action, research and critical reflection, while engaging all stakeholders in the research process. Action research thereby enabled a broad understanding of complex processes in practices and enhanced the applicability of our study outcomes [29, 30]. The action research cycle consisted of qualitative (semi-structured interviews) research, incorporating the perspective of both custodial staff and management as well as youth and their parents. This was accompanied by an iterative validation, feedback and reflection loop. As outlined above, the duration of the pilot varied per facility, with Amsterdam running the longest; therefore, it was monitored most intensively.

The current study is embedded in a broader evaluation the facilities: the research team carefully monitored all aspects of the three facilities from the process of screening and indication for placement to follow-up a year after release. This broader evaluation significantly contributed to the theoretical sensitivity of the research team: the level of insight of researchers in the phenomena under study and the context in which it emerges, how attuned researchers are to the nuances and complexity of participant’s words and action and their ability to reconstruct meaning from data [31]. Hence increasing the validity and practical applicability of the results.

### Semi-structured interviews: sample

In determining our sample, a comprehensive method of source triangulation was applied to increase validity of the results. Our research included the three sites of the pilots facilities (Nijmegen, Groningen and Amsterdam) and our sample constituted three groups of stakeholders: staff, youth staying in the facilities and their parents. During the evaluation period – between September 2016 and November 2019 – a total of 63 semi-structured interviews were conducted.

#### Staff

Eighteen interviews were conducted with staff working at the pilot locations in three rounds: in May 2017, December 2017 and in October 2018. Staff were sampled through purposive sampling to ensure they would represent different job levels within the participating organizations. In the first round, the behavioral expert (responsible for the assessment and planning of youth’s trajectories and management of unit staff) of each pilot facility was interviewed (*n =* 3*)*. The second round consisted of a group interview (*n =* 3*)* at each location with the behavioral expert and representatives of the unit staff from each location; in a consensus building process, the group interview allowed for a more in-depth investigation of the themes emerging from the first interviews. In the last round, the entire unit staff (with the exception of one staff member, who was on leave) of the Amsterdam facility was interviewed: including security staff (*n* = 5) and social workers (*n =* 7). Some of these staff members had previous experience working in a secure setting with an emphasis on physical and procedural security; allowing them to put their current experiences into that perspective. All staff who were approached to participate, agreed to do so.

#### Youth

During the evaluation period, 204 youth were remanded or sentenced to one of the three facilities: 20 in Nijmegen, 28 in Groningen, and 156 in Amsterdam (these numbers differ as the duration of the pilot differed between the facilities). Of this total sample, 35 youth were approached to participate, resulting in 29 interviews: seven in Nijmegen (one declined), seven in Groningen (two declined), 15 in Amsterdam (three declined). To ensure youth were somewhat adjusted to the facility and familiar with the facilities policies and procedures, they were approached to participate after the first week of custody. Further, a fair share of the participating youth had previously been placed in a secure setting with high levels of physical and procedural security; allowing them and their parents to put the current experience into that perspective.

Sample selection was guided by FS, through a combination of availability sampling (guided by which youth were placed in the facility at the time of the interview rounds), convenience sampling (guided by the process of data collection and analysis), and purposive sampling to ensure a heterogeneous sample, representing the diversity of the total sample. The *age* of participating youth varied between 15 and 24 years (mean = 17.48, SD = 2.15); in the total sample the age varied between 13 and 24 (mean = 16.29, SD = 1.7). One *girl* and 28 *boys* were interviewed; the total sample included one girl. In the participating sample 24 youth (83% participants) were detained *pre-trail* and five (17%) were *post-trail*; in the total sample respectively 94% and 6% of youth were detained pre- or post-trial. In the participating sample youth were suspected or convicted of a property (19%), violent (18%), or violent property (63%) *crime* (in the total sample these percentages were respectively: 22%, 21%, 52%). The *duration of stay* of the participating youth varied between 13 and 213 days (mean = 80.22, SD = 57.52); in the total sample the duration of stay varied between 2 and 213 days (mean = 43.51, SD = 41.99). Of the participating youth two (7%) had a *preterm exit*; in the total sample this was 14%.

#### Parents

Of the 29 interviewed youth the involved parent (or caregiver) was approached to participate in the research (convenience sampling). We only approached the parents if we obtained permission from youth: in eight cases youth objected so we approached 21 parents. This resulted in 16 interviews (three in Nijmegen, five in Groningen and eight in Amsterdam), in most cases with the biological mother. Reasons for parents not to participate varied: three parents could not be reached by the research team, one parent did not agree to participate, and in one case the parent did not speak Dutch while an interpreter was not available.

### Semi-structured interviews: data collection and analysis

A trained and supervised research team, including author FS, conducted the interviews. All participants were personally approached by a member of the research team explaining the nature and objective of the study. The interviews lasted approximately one hour and were semi-structured by a topic-list (available upon request by the corresponding author). As the study was part of a larger research project, during the first round of interviews a large number of topics were discussed. The topics relevant for this paper focused on: the overall institutional climate, the alliance between staff and youth, the dynamic amongst youth, security measures, definition of relational security and working mechanisms, staff responses to misconduct and violence, participants safety experience. As specific factors emerged from the data the concurring themes were highlighted in the interviews for more in depth investigation. New emerging themes, that were not anticipated with the initial topic list, were followed up on in subsequent interviews.

All interviews were tape recorded, transcribed verbatim and uploaded into MAXQDA. The verbatim of each interview was coded employing a method of ‘thick analysis’ [32] including open- and causal coding. Through an inductive analysis these codes were sorted, interrelated and grouped to build categories of the conceptualization of relational security; carefully noting the specific aspects and the direction of association in memo’s for a cross check of causality. In the second phase – intermediate coding – fully individual categories were formed by connecting sub-categories that reflect the properties and dimensions of the different categories. As the most advanced form of this intermediate phase, an axial analysis was applied to integrate categories. Each round of coding and subsequent analysis was performed through constant comparison [33] between codes, between categories, within each interview, between participants, and between the three sites. In the last round of interviews data saturation occurred [33]: no new themes emerged from the participants’ narratives in subsequent interviews.

### Validation, feedback and reflection

Validation, feedback and reflection took place through peer debriefing, onsite observations and member validation. These activities improved the validity and reliability of the results and their applicability in practice [32]. First, each step of data collection, analysis and reflection was carefully noted in memo’s, combined in a logbook by the principle investigator (FS) who conducted the analysis. Throughout the duration of the study this logbook was discussed with a senior researcher (EM) through peer debriefing [32]. In general, there was agreement between the researchers on the results and interpretation. Second, about three weekly onsite observations involved the researchers spending a day at the facility interacting with youth and staff, which allowed the research team to develop a good understanding of the research setting and daily practices. Observations were written down in structured field notes, focusing on the same topics as the interviews. These observational data were used to contextualize and supplement the interview data. Third, as part of the pilot, representatives of local and national government and managers and practitioners from relevant organizations met every six weeks to discuss the pilot’s progress and formulate actions. Member validation [34] was applied through these periodic pilot’s advisory board meetings after each cycle of data-collection and analysis. Apart from some linguistic modifications, no major changes were suggested by staff at the advisory board meetings, indicating good validity and practical applicability of the outcomes of this study.

## Results

The interviews provided coherent results between participants (staff, youth, parents) and sites (Amsterdam, Nijmegen, Groningen) in answer to the two research questions. Table 1 provides a summary of the results (each aspect is described in detail below).

**Table 1:** Summary of results

1. How is relational security conceptualized in a youth justice setting?
1.1 Basic attitude staff: connection with and attunement to each individual youth 1.2 Constructive alliance between staff and youth 1.3 Staff presence
2. How does relational security contribute to safety in a youth justice setting?
2.2 Staff physical presence: prevention, intervention and support 2.3 Staff’s insight and understanding of youth 2.4 Youth’s (self-)insight 2.5 Youth’s motivation to take responsibility and empowerment 2.6 Positive interactions between staff and youth 2.7 Positive interactions between youth 2.8 Constructive institutional climate 2.9 Relational security promotes positive youth development

### How is relational security conceptualized in a youth justice setting?

According to staff, youth and their parents, relational security is grounded in three distinct, but interrelated, elements: (1) staff’s basic attitude directed towards connection with and attunement to each individual youth, (2) constructive alliance between staff and youth, (3) staff presence.

#### Basic attitude staff

Staff’s basic attitude concerns the way staff shape interactions with youth. The core of this attitude is the connection with and attunement to each individual youth and their context; and knowledge and insights about youth to be able to see things from their perspective and logic. The way staff interact with youth is tailored to each individual based on their needs and strengths. The professional accepts the young person as he/she is, gives youth space to be themselves, and shows genuine interest in getting to know youth beyond their crime and case file (‘don’t judge a book by its cover’). Hereby staff mention that they try to be constantly aware that certain behavior (e.g. impulsivity, risk-taking, rule breaking) is to some extent ‘normal’ behavior as part of adolescent development. *Staff: “Safety comes down to relational security. That is: making youth feel that they are important, that they matter, that you want to be there for them and invest in them. That you see the good in them. They have done bad things, but they are not bad children. Youth feel that we care about them. For example, if they arrive late from leave I first ask if they are ok instead of immediately telling them of. I think you achieve a lot with that. I believe that this approach makes that they don’t abscond, because they feel welcome here.”*

Another aspect is staffs ability to show respect for personal boundaries. On the one hand, the professional must respect the personal boundaries of youth; not force contact and a relationship and allowing youth the space to withdraw and cool off when emotions run high. On the other hand, the professional must set clear personal boundaries and make youth aware of their behavior and consequences if these boundaries are violated. Rather than strict protocols, the personal norms and values of a professional are brought more to the forefront. This means that difference exists between staff in where boundaries are set. According to staff and youth this is workable if professionals acknowledge that these differences exist and take the time to explain why they set a certain boundary, when there is overall clarity about rules that are set in stone, and when youth experience that this respect for personal boundaries is reciprocated. *Staff: “I don’t like it when someone is constantly breathing down my neck. So why would I do that to someone else. Especially to youth you barley know.”*

The professional basic attitude is further shaped by: i) Warm care (creating a warm welcome, actively offering youth care and attention, seeing and caring for ‘the child’ behind ‘the criminal’); ii) Sincerity (showing ‘the person’ behind ‘the professional’, showing vulnerability and honesty); iii) Empowerment (complimenting and rewarding things that go well, expressing trust explicitly, actively motivating youth, formulating small positive steps together with youth); iv) The professional as a role model (at all times staff ‘practice what they preach’); *Parent: “These youth need love and attention. They go down the criminal path, but essentially deep down they’re not like that. It is also a form of seeking attention and care, which they often lack.”*

#### Constructive alliance

A constructive collaboration between staff and youth is characterized by four aspects. First, youth (and their parents) are considered co-owner of their case plan and get a seat at the table to be actively involved in the planning and evaluation of their trajectory. Youth are also involved in developing institutional policies. Youth’s perspective, goals, wishes and skills are centralized in this. Staff have an open and transparent way of working toward youth and their network. Second, the young person is given autonomy and room to take responsibility (in line with youth’s capacities). Within the context of relational security staff divert from a strong risk-focused to a more strengths-based approach. Third, youth get the space to learn by trail and error. This is built on the principle that behavioral change hardly ever occurs in a linear process. A violation of the rules or agreements is considered to be a moment for learning and reflection. Youth are included in the settlement and the determination of an appropriate consequence tailored to their needs; whereby the focus lies on restoration, the underlying causes of the behavior and what is needed to prevent similar situations in the future. Finally, staff stand next youth as a coach and support them wherever necessary. If needed, clear boundaries are set, but staff’s attitude is more advisory and motivating than repressive and authoritarian. Participants stress clear boundaries are key in a safe environment. This, however, does not necessarily mean staff can’t allow flexibility and negotiation on boundaries. It means staff collaborate with youth and are consistent and transparent in their approach to decision-making, guided by a careful weighing of youth’s individual needs. *Staff: “We learn by mistakes. Especially these youth: they are children, they are still developing. If I make a mistake I much rather have someone talk to me about it. So I can learn how to prevent it in the future. Instead of punishing and belittling me, diminishing my self-esteem. This works the same for these youth. With punishment you create distance.”*

The participants stress that justice-involved youth generally tend to distrust people and keep staff at a distance. Building a relationship of trust takes a long time and requires intensive and continuous contact. At the same time, safety must be immediately guaranteed. Staff express that the constructive alliance is thus not (directly) about forming a deep trusting relationship, but about cooperation based on reciprocity, sincerity and transparency; and confidence of both parties that the other will adhere to the mutual agreement. *Staff: “These youth per default don’t trust you. They look at what you can do for them. If you show them that you can mean something for them and try to understand them. Then you might at one point achieve their trust. It starts with being reliable.”*

#### Staff presence

The final element of relational security relates to staff being actually physically present. This aspect concerns staffing numbers (youth/staff ratio) and the time and space available for face-to-face contact. Instead of spending most working hours in an office, even if this office is linked to the communal areas, staff spend most working hours in communal areas. *Youth: “Being safe is not about the building, it is about the people around me.”*

Youth hereby stress the importance of a good balance between supervision and trust. They highlight the importance of staff being present, but don’t want to feel like staff is constantly watching them every move as a subject of suspicion. *Youth: “If staff are constantly looking at you expecting that you will do something bad this creates tension. This makes you feel fucked-up.”*

### How does relational security contribute to safety in a youth justice setting?

All participants reported that they experienced the facility as a safe environment and stated that there were very few violent incidents. Also, staff expressed high job satisfaction. *Parent: “For me it is very important that safety is guaranteed. As a mom I feel like he is in a good place there [at the facility]. Staff provide safety and take good care of him.” S*taff, youth and their parents identify several mechanisms through which relational security as the core of the security framework contributes to safety, which are outlined below (see Table 1 for an overview).

#### Staff physical presence: prevention, intervention and support

By being physically present – in a constructive supportive way – staff promote safety through: the preventive effect based on the presence of the professional; and by observing, signaling, intervening early and de-escalating if necessary. *Youth: “Small incidents don’t escalate because there is always someone there.”* Further, this way, staff are available for youth to seek (emotional) support if needed; and it creates many opportunities for informal contact between staff and youth (e.g. playing a video game), which contributes to positive relationships and a positive institutional climate.

#### Staff’s insight and understanding of youth

Through the three elements of relational security, staff experience that they are able to connect with youth, understand them with regards to their triggers, needs and strengths, and act appropriately on the basis of this insight to guarantee safety. *Staff: “It is important to connect with youth to figure out the underlying issues that lead to certain behavior and uncover their way of thinking and way of life. You cannot derive this only from a casefile; you need to hear it from youth themselves. Contact and connection with youth is essential.”*

#### Youth’s (self-)insight

The collaborative way of working also promotes self-insight in youth into their own thought- and behavioral patterns, where risks and opportunities lie for them, and what is needed to achieve their goals. Further, staff show youth how boundaries can be set and conflicts can be dealt with in a non-violent manner. These insights obtained by youth reduce the likelihood of (re)occurrence of undesirable behavior. *Youth: “Here you are confronted with your behavior. In a prison they just put you in seclusion when you have done something wrong. Here I found out a lot of things that I didn’t expect to find out about myself.”*

### Youth’s motivation to take responsibility and empowerment

Participants stress that being deprived of your liberties is very stressful in itself, but because at the facility youth are still given a certain level of autonomy this takes a ‘certain pressure of’. Because youth are given a certain degree of autonomy and staff continuously motivate them to take responsibility, they feel more responsibility to promote a safe environment and they are more motivated to take that responsibility. All participants highlight that the key to motivation lies in active collaboration with youth and positive reinforcement. A collaborative way of working fosters a sense of shared ownership and promotes youth’s support for the agreements that are made and the consequences that follow after violation of these agreements. Safety is further promoted by the fact that relational security provides a way of working that empowers youth and gives them the confidence that they actually can handle this responsibility and achieve their goals. *Youth: “You really see that they give you a chance, either you’re going to do good yourself or you’re going to screw it up yourself. They give you the space to show that you want to do well, but they also give you the space to fuck it up. You can walk right out the door, there is no one to stop you. It’s your choice if you go, you go. I have been at a tipping point to do stuff, because my instincts tell me. I’m very impulsive. Yet the thing is, I’m reminded of the fact that it is better to not do those things.”*

On the other hand staff and youth stress that secure settings with an overreliance on physical and procedural security creates false security as it crumbles youth’s motivation to comply to the rules. *Youth: “Will you also share these results with the minister? Look we pay a lot of taxes in this country and they [the government] spend money like it is nothing on prisons. But high walls and big doors are a waste of money. If I want to break the rules I will.”*

### Positive interactions between staff and youth

All participants stress the importance of reciprocity: within the interplay between staff and youth, staff get back what they put into it. *Youth: “It is all about how staff act towards us. If they are relaxed, we are relaxed.”* Through relational security staff interact with youth in a constructive and respectful manner. Youth reciprocate this, which promotes a positive relationship between staff and youth and reduces the risk of conflict. Also, as relational security involves a non-violent way of working and staff refrain from authoritarian behavior, it has a de-escalating effect on situations that (potentially) jeopardize safety. Participants further experience that relational security promotes a dynamic between staff and youth that is less of the ‘us vs. them’ dynamic generally seen in youth justice facilities. *Youth: “They [staff] are focused on you individually, your development. This is different inside [other youth justice institutions]. There they have more standard sanctions, this or that. It is more them vs us.”*

### Positive interactions between youth

Relational security promotes constructive dynamics amongst youth, according to the participants. The risk of undesirable group formation between youth or the explicit teaching of deviant behavior by group members appeared to be reduced by relational security. Relational security involves a way of working that is tailored to each youth’s individual risks and needs, without a strong group-based approach. Youth and staff state that with this way of working youth were more focused on themselves and their future, rather than on the other youth and their position within the group. *Youth: “It [violence] has not happened here. Because everyone is focused on themselves. I can’t be bothered to focus on other people’s business.”*

### Constructive institutional climate

On the institutional level participants state that relational security as the core of the security framework, with low levels of physical and procedural security, contributes to an constructive institutional climate (i.e. the quality of the social- and physical environment) that makes youth less inclined to display behavior that jeopardizes safety. For example, staff and youth express that youth are less inclined to abscond because they feel that the facility offers them a positive homely environment and the support they need. *Staff: “It is safe here because we are available for youth if they need support. We stand next to them instead of above them. Of course we also set boundaries, but we don’t carry out all day that we are ‘the boss’. They experience that we genuinely enjoy working with them, doing things together. Youth sense this positive vibe.”*

### Relational security promotes positive youth development

Finally, staff, youth and their parents note that relational security creates a setting that promotes positive youth development. Relational security, combined with relatively low levels of physical and procedural security, fosters a setting that is “as normal as possible” and youth do not experience isolation from society. Participants stress that this security approach allowed youth to maintain and build on protective factors outside the facility (like positive social ties, work or education). Youth’s parents experienced that this way of working permitted them to be more easily involved during the time of confinement and maintain their role as caregiver. Participants note that by all of this, relational security – as opposed to a strong reliance on physical and procedural security – likely contributes to reducing the risk of re-offending. *Youth: “Ok so, look at it this way. You are sick and I want you to get better. Then I throw you into a room full with sick people. Do you think you will get better? Hell to the no. That is why I believe this approach works. Why? Here I am surrounded with positive people, healthy people. I take something positive with me when I leave; staff inspire me.”*

## Discussion

Based on action research the current study provides a conceptualization of relational security for youth justice facilities. We found that relational security is grounded in three distinct, but interrelated, elements: (1) staff’s basic attitude directed towards connection with and attunement to each individual youth, (2) constructive alliance between staff and youth, (3) staff presence. These elements complement and reinforce each other and all three need to be in place.

This study further provides insight into how this way of working promotes safety as experienced by staff, youth and parents. Relational security - as the core of the security framework - promotes safety through several mechanisms. By being physically present staff foster safety through prevention and by deescalating if necessary. Relational security further promotes staff’s insights and understanding of youth to act appropriately on the basis of this insight; and enhances youth’s (self-)insight on risks and strengths. Because youth are given a certain degree of autonomy and staff continuously motivate them to take responsibility, they feel more responsibile to promote a safe environment and they are more motivated to take that responsibility. Through relational security staff interact with youth in a constructive and respectful manner. Youth reciprocate this. Also, staff, youth and parents experienced that relational security reduces the risk of undesirable group formation between youth or the explicit teaching of deviant behavior by group members (deviancy training). Because relational security contributes to a therapeutic institutional climate youth are less inclined to display behavior that jeopardizes safety. The results show that each of these mechanisms works both ways. For example, when staff *do not cooperate* with youth, youth show *more resistance* towards the rules. Relational security turns the ‘aggression-coercion cycle’ around, promoting constructive behavior in youth *and* staff. Finally, relational security promotes important developmental competencies like autonomy; and builds positive identities through positive relationships. Effective relational security - combined with low levels of physical and procedural security - not only safeguards staff and youth within the facility, but also provides an approach to support youth to establish safe connections with their family and community and foster positive development and a life away from crime.

### Methodological considerations: limitations and strengths

Two methodological considerations are worth noting for the interpretation of these results. First, even though maximum diversity in the sample of youth and parent participants was sought, the study relied on their willingness to participate. All professionals who were approached for the study agreed to participate, but a few youth and parents declined. Youth and parents who were not willing to participate could have had a different view and experiences. Second, member validation only involved staff. The results were derived from constant comparison between participants groups finding similar outcomes; and no major changes were suggested by staff. However, member validation would have preferably also involved youth and parents.

Notwithstanding these remarks we consider this study to be methodologically robust: as outlined in the method section multiple procedures were followed to promote the validity and reliability of the results and their applicability in practice. Our conceptualization of relational security reflects the collective learning of staff in developing this way of working and the first hand experiences of youth and their parents. To our knowledge this is the first empirical study exploring relational security through action research in the context of a youth justice facility with relational security at the core of its security framework. This study provides an important contribution to filling existing knowledge gaps. Below we outline existing theories that substantiate our results and implications for research and practice.

### Underlying theories

Our conceptualization of relational security finds support in the well-established literature covering the therapeutic alliance (as outlined in the introduction) and can be substantiated by two aligning youth justice theories. First, the conceptualization fits within a social-ecological theory and vision of youth offending [35]. This theory contrasts the dominant ‘risk-based models’ like the Risk-Need-Responsivity model [36] and instead focusses on strengths and positive youth development. Drawing on Bronfenbrenner’s ecological system model of development [37]: “a social-ecological perspective decenters the young person as the source of the offending problem, seeing them in terms of the relationships, interactions and processes that define and influence their everyday lives and experience. This perspective recognizes the importance of regular, deep interactions, meaningful to the child – and that new interactions can effectively alter development (or the direction of development) and therefore influence outcomes such as behaviors.” [35: page 7]. This theory sees youth not as ‘problems to be managed’ but ‘resources to be developed’ [38]. A relational strengths-based approach characterized by the ‘five Cs’ – competence, confidence, character, connection and caring [38] will promote positive development [35]. These C’s are directly reflected in the conceptualization of relational security.

Our conceptualization of relational security also corresponds with Self Determination Theory [39], which complements the social-ecological theory and recognizes similar concepts. Self Determination Theory focuses on the social-contextual conditions that facilitate or undermine the processes of self-motivation and healthy development. The theory stresses the importance of three innate psychological needs: competence, autonomy and relatedness, which when satisfied promote treatment motivation, mental health and resiliency [39, 40]. The involuntary nature of youth justice settings, particularly when there is a strong emphasize on physical and procedural security, automatically undermines these needs and thereby youth’s treatment motivation [40]. Relational security provides an approach that explicitly recognizes and promotes competence, autonomy and relatedness as much as possible within the boundaries of the secure setting. And thereby – as experienced by staff, youth and parents – enhances youth’s treatment motivation and positive development.

### Implications for practice and research

To further broaden our knowledge on relational security several implications for research and practice can be derived from our results. Overall, given the complex environment of a youth justice facility, relational security is best explored by a multi-perspective approach and a combination of qualitative and quantitative research, on the intersection of research and practice. We therefore discuss the implications for research and practice combined on six main topics that were derived from our results: transferability of the results; relational security as the core of the security framework; the interplay between relational security and other security measures; the institutional context; relational security vs. dynamic security; and limitations of relational security.

#### Transferability of the results

First, the youth justice population is heterogeneous. The current study covers a selection of this divers population: before placement in the facilities risks and needs are assessed to determine the appropriate level of security and care (See Souverein et al. [27] for a detailed description of this process). Also, during placement youth can be transferred to facilities with higher levels of physical and procedural security after severe rule-breaking. It is important to explore how this conceptualization of relational security transfers to other subgroups, taking gender into account. During the course of the current study only one girl was placed in the facilities. She was included as a participant and her interview did not yield any outstanding results. Though other research suggest that girls, generally speaking, might be more sensitive to the institutional climate and their interaction with staff [16]. Research should explore *if* and *how* gender-differences exists on the interpretation and impact of relational security.

Considering the above, one may question whether all results will transfer from this specific context to other settings: how relational security is shaped in different secure residential settings for youth within and across different sectors (mental health, welfare, justice)? At the same time other studies suggest that the underlying principles of relational security generally align with youth’s experiences of safety and security across residential settings. A recent study in the Netherlands [41] – across residential youth care facilities in different sectors and with various levels of security – explored how youth define institutional ‘safety’ and what factors contribute or hinder their experienced safety. Combining quantitative and qualitative methods this study found similar results across settings. Youth define a safe setting, in which they can develop themselves, as a place where: staff take them seriously, they can discuss any matter with staff and they experience that staff truly listens. This sense of safety is promoted when youth experience: a sense of autonomy and agency; a connection with staff; staff intervene to deescalate situations that (potentially) jeopardize safety; fair and clear rules and routine; and they can reach out to their support network (family or friends) if they need. These results align with our conceptualization of relational security. This speaks for a wide applicability of this concept in secure residential facilities for youth, with possibly some nuances for specific subgroups (e.g. gender). Practice, accompanied by action research, should further explore this.

#### Relational security as the core of the security framework

In line with the previous point our results highlight the importance of a shift in focus from a strong reliance on physical and procedural security to relational security as the core of the security framework. This also leads to the question whether is it always necessary to raise the levels of physical and/or procedural security when safety is jeopardized or that staff may also rely on intensifying the levels of relational security to restore safety. In the current context, as mentioned above, during placement youth could be transferred to facilities with higher levels of physical and procedural security. A case study of these transfers revealed that, in most cases, these youth did not necessarily need more intensive levels of security. Instead, safety was jeopardized because these youth had more intensive and specialized care needs [27]. The facilities required a certain level of independence that wasn’t suited for all youth (e.g. for some youth with a mild-intellectual disability). A more intensive level of relational security, which is about providing care to establish security, could possibly have provided a solution in these cases, instead of raising the levels of physical and procedural security. Relational security can be an intense security measure: it is inherent to the way of working that behavior of youth lies under a magnifying glass and staff may intensify their interactions with youth. Practice-based research should explore how we may differentiate relational security on the spectrum of low to high intensity levels of security. And how secure settings can be supported to resist the common practice of automatically raising physical and procedural security when safety is jeopardized.

#### The interplay between relational security and other security measures

The results reveal that the security framework is a systemic model: every dimension (physical, procedural, relational) is inter-related, directly or indirectly, such that modification of one dimension may affect the others. All three security dimensions need to be in place, but it is important to find the right balance between physical, procedural and relational security [42]. Given their interrelatedness, research should focus on further uncovering the dynamics between these three dimensions – in settings that are grounded in relational security.

#### Institutional context

The security framework in turn exists within the broader institutional context. Our results reveal that relational security requires specific professionalism. For example, personal norms and values of staff are brought more to the forefront, rather than strict protocols. This requires (self)reflective and metalizing capacities. Also, the fact that youth and parents experienced the facility as a ‘homely environment’ seems to contribute to effective relational security. The influence of the organizational factors is also found in other research. In a qualitative study of a conventional youth justice facility – with a strong reliance on physical and procedural security and little relational security – staff identified several environmental aspects that impacted upon their interactions with youth and their ability to maintain safety [42]. Staff, for example, stated that the lack of private spaces and prison-like design characteristic of the facility (e.g. lack of daylight and green spaces) were barriers for relationship building and establishing a therapeutic climate. Also the large unit size (15 beds) felt ‘unmanageable’ and, according to staff, directly contributed to youth’s behavior escalating more frequently. Other studies have also highlighted the impact of the unit size on institutional safety [43]. While there is growing recognition that the physical environment of a facility impacts the relationships between staff and youth, there is a dearth of research in this area [42]. A comprehensive understanding of relational security and it’s potential in youth justice settings requires more practice-based research on the institutional factors that facilitate or hinder relational security.

#### Relational security vs. dynamic security

In research and practice the terms *relational* security and *dynamic* security are often used interchangeably. It seems, however, that these terms have different origins. Relational security emerged in the forensic mental health literature [44]; whereas dynamic security originated in prison settings [45]. It would be interesting to explore if these terms underlie similar mechanisms or describe inherently different constructs given their origin.

#### Limitations of relational security

Finally, the current study focusses on the potential of relational security. Obviously, establishing relational security within the complex setting of youth justice facilities comes with challenges and dilemma’s. And it may also have its limits. It is important for research and practice to also explore this side of the medal.

## Conclusion

Our results provide practitioners, policy makers, and academia with an understanding of what relational security is and how it effects institutional safety in a youth justice setting, particularly a small-scale community-embedded facility. Relational security can be defined in a practical concept; outlining a way of working that guides staff in how to establish a safe and therapeutic environment in residential forensic youth care. This conceptualization of relational security finds support in existing theories and methods. It contrasts the conventional approach of youth justice facilities to foremost rely on physical and procedural security. When we consider the developmental vulnerability of justice involved-youth [46, 47] youth justice is not only a matter of responding to harm caused *by* youth, but also of addressing and working to counteract harms *to* youth. When a youth justice system responds through predominantly punitive and risk focused means, this constitutes a form of violence against youth, albeit legally legitimized violence. Relational security is not only *a way of working*, but also *a way of being.* It encompasses a vision about security and mentality towards justice-involved youth that sees them not merely as ‘risks to be managed’, but as young people in the prime of their development acknowledging their strengths and opportunities and allowing them to learn from mistakes. Through the lens of relational security ‘care’ and ‘security’ are not viewed within the paradox – establishing security *or* providing care – often found in conventional prison-like facilities. Relational security is about *providing care to establish security*. An approach to security with great benefits and potential according to staff, youth and their parents.

## Data Availability

Due to ethical concerns and the sensitivity of the data, supporting data cannot be made openly available. Information about the data and conditions for access can be discussed with the corresponding author upon request.
